# Secretory Activity in the Longitudinal Canal of Male *Podarcis siculus* Lizards

**DOI:** 10.3390/ani16193057

**Published:** 2026-09-28

**Authors:** Salvatore Desantis, Antonio Ciro Guaricci, Mario Cinone, Maria Albrizio

**Affiliations:** Department of Precision and Regenerative Medicine and Ionian Area (DiMePre-J), University of Bari Aldo Moro, 70124 Bari, Italy; antoniociro.guaricci@uniba.it (A.C.G.); mario.cinone@uniba.it (M.C.); maria.albrizio@uniba.it (M.A.)

**Keywords:** reptiles, reproduction, testis, rete testis, efferent ducts, epididymis, histochemistry, ultrastructure, secretion, Squamates

## Abstract

In lizards, the longitudinal canal conveys the sperm from the rete testis to the ductuli efferentes, which connect to the cranial end of the epididymal duct. Due to its reduced extension, the longitudinal canal has been largely ignored compared with the ductuli efferentes and the ductus epididymis. The objective of this study was to provide information on the structure and function of the longitudinal canal in the lizard *Podarcis siculus* by using light and transmission electron microscopy. For this purpose, testes and epididymides from adult male *P. siculus* lizards captured during the reproductive period were used. Light microscopy showed that the longitudinal canal was lined by a simple squamous or low cuboidal epithelium containing apical periodic acid-Schiff (PAS)-positive granules. Transmission electron microscopy revealed two types of epithelial cells: ciliated and non-ciliated. The non-ciliated cells showed electron-dense granules in the supranuclear and apical regions, suggesting probable secretory activity. These findings demonstrate, for the first time, that the longitudinal canal of the lizard *Podarcis siculus* is not merely a passageway for the sperm but may play a role in the composition of the medium in which these gametes are found during their flow towards the epididymis.

## Simple Summary

The male reproductive system of the lizard *Podarcis siculus* also includes the epididymal longitudinal canal, which connects the rete testis to the outer edge of the cranial zone of the epididymis. This canal has been considered a simple passageway for sperm from the testis to the epididymis. Using histochemical and ultrastructural investigations, we observed that the epithelium lining the longitudinal canal produces secretory granules. This suggests that this extratesticular duct may influence the chemical composition of the luminal environment through which sperm pass, with possible effects on the molecular composition of their surface as they move towards the epididymis. Therefore, the likely active role of the longitudinal canal in the male reproductive physiology of *Podarcis siculus* must be taken into account.

## 1. Introduction

The family Lacertidae includes more than 250 species with a wide geographical distribution [1]. The Italian wall lizard, formerly known as *Lacerta sicula*, then *Podarcis sicula*, and currently *Podarcis siculus* (Rafinesque-Schmaltz, 1810) (Sauria, Lacertidae), is a widely distributed species in Mediterranean countries [2]. *P. siculus* is not listed as threatened by the International Union for Conservation of Nature (IUCN) [3]. This species was introduced to New York in the late 1960s [4], and it is present in a Natural Ornithological Park in Russia [5]. *P. siculus* is the most abundant species among the reptiles of southern Italy. The possibility of keeping animals, even for extended periods, in terrariums has made this lizard an interesting experimental model for studying the biology of reptiles. In more recent years, *P. siculus* has also been used as a biological model in male reproductive toxicology [6].

Like other *Lacertidae*, *P. siculus* is an ectothermic species, and sexual dimorphism becomes particularly evident during the reproductive season, when males develop femoral pores. The reproductive annual cycle of this species is well documented by morphological and endocrinological studies [7,8,9]. Its gametogenesis is under the control of the hypothalamus–pituitary–gonadal axis, which is regulated by photothermal regimes. In *P. siculus*, the breeding period occurs between late April and mid-July. This ensures that young emerge in summer, when environmental factors, such as temperature and food supply, are favourable. In the male, complete spermatogenesis occurs between late April and June. After mating, the animals enter a refractory period (summer stasis) (July–October), during which they are unresponsive to endogenous or exogenous hormones or temperature manipulation. Spermatogenesis begins in autumn (resumption phase), when only a few sperm are produced, which are never able to reach the epididymis; therefore, they are not useful for reproduction. This period is followed by the winter stasis. In spring (March-April), the resumption of testicular activity (spring resumption) occurs. This represents a preparatory phase for the following reproductive period, in which there is intense spermatogenesis and spermiogenesis.

In squamate reptile males, the accessory ducts of the testis consist of the rete testis, ductuli efferentes, efferent ducts, ductus epididymis, ductus deferens, and ampulla ductus deferentis, which convey sperm to paired hemipenes [10,11,12,13,14].

The ductulus efferentis represents the mammalian extratesticular rete testis on developmental grounds, as it is derived from the rete blastema. The efferent ducts and the epididymal duct are derived from mesonephric tubules [13,15].

Several reports demonstrate that the number of ductuli efferentis exiting a testis varies among reptiles, regardless of taxonomic classification (see [16] for references).

Regarding *Lacertidae*, a single ductulus efferentis connects the testicle to the cranial zone of the epididymis. More precisely, the ductulus efferentis opens into the longitudinal canal that gives rise to several efferent ducts (the number varies depending on the species); these, in turn, may empty into the epididymal duct, which continues as the duct deferens [17,18,19] (Figure 1). The longitudinal canal runs along the outer edge of the epididymis [19]. The presence of the epididymal longitudinal canal has also been found in the Anguidae *Anguis fragilis* [20], Scincidae *Plestiodon fasciatus* [21] and *Scincus scincus* [22].

Histological investigations of the epididymal longitudinal canal are very limited. It has been reported that the longitudinal canals of *Podarcis muralis* [18], *Scincus scincus* L. and *Chalcides ocellatus* [18,22] have a flat, non-ciliated epithelium. However, to date, no histological study has been conducted on the longitudinal canal of the lizard *P. siculus*, in contrast to the ductuli efferentes [23] and the ductus epididymis [24] in which histochemical and ultrastructural investigations have been conducted. Regarding the other Squamates that have a longitudinal canal, simple histomorphological analyses have been carried out on the epithelium of this canal in *Plestiodon fasciatus* [21] and *Scincus scincus* [22].

The extratesticular environment through which sperm move can, to some extent, affect their maturation. In this study, we present, for the first time, histological, histochemical and ultrastructural findings for the longitudinal canal of *P. siculus*, with the primary objective of determining whether this canal is simply a passageway for sperm or whether it contributes to the composition of the environment that the sperm interacts with before reaching the efferent ducts, thereby playing a functional role in the reproduction of this lizard species. To this end, this study was conducted on adult male P. siculus during the reproductive period. This study could provide better insight into the *P. siculus* male reproductive system and more generally into the male reproductive system of reptiles.

## 2. Materials and Methods

### 2.1. Animals

Adult males (*n* = 4) of *Podarcis siculus* were captured during the reproductive period (May–June of 1987–1988) in the neighbourhood of Bari (Southern Italy; Latitude: 41°07′31″ N; Longitude: 16°52′00″ E). After capture, the animals were transported to the Institute of Zoology (University of Bari), anaesthetized with ether (diethyl ether) and euthanized by decapitation. From each lizard, the entire testis-epididymis complex was removed from one side, while the testis and epididymis of the other side were separated from one another. The testis-epididymis complex was processed for light microscopy, while the separated testis and epididymis were fragmented and processed for transmission electron microscopy (TEM) investigations.

### 2.2. Light Microscopy

#### 2.2.1. Morphological Analysis

The specimens destined for light microscopy studies were fixed in Bouin’s solution for 24 h at room temperature. Then they were dehydrated through a graded series of ethanol and embedded in paraffin wax. Serial sections (5 μm thick) were cut. After dewaxing with xylene and hydration in a descending ethanol series, they were stained with Hematoxylin-Eosin (H&E) or with Galgano trichrome for general morphology and with histochemical procedures for glycan characterization. In addition, histochemical analyses were performed using the combined Alcian blue pH 2.5–periodic acid-Schiff (AB 2.5-PAS) methods.

#### 2.2.2. Glycohistochemistry

For glycan characterization, sections were treated with (1) the periodic acid-Schiff (PAS) reaction that stains neutral glycans magenta and (2) Alcian Blue pH 2.5 (AB 2.5) that stains acidic (carboxylated and sulphated) glycans blue. The mixtures of neutral and acidic mucins appear as purple staining [25].

#### 2.2.3. Morphometric Analysis

The height of lining epithelium and the width of the longitudinal canal were determined using 10 microphotograph fields randomly selected and taken with a digital camera (DS-U3, Nikon, Tokyo, Japan) connected to an Eclipse Ni-U light microscope (Nikon, Japan), using a 100× objective. The images were analyzed by the image-analyzing program NIS Elements BR (Vers. 4.20) (Nikon, JP). For the measurement, cells in which the plane of the section clearly passed through the cell nucleus, parallel to the longitudinal axis of the cells, were selected. All values were expressed as mean ± standard deviation (S.D.).

### 2.3. Ultrastructure

Fragments (about 5 mm in diameter) of the cranial part of the epididymides were fixed in 4% glutaraldehyde buffered with 0.1 M sodium cacodylate (pH 7.2) for 4 h at 4 °C. After a rinse, tissues were post-fixed in 1% OsO_4_ buffered with sodium cacodylate for 2 h at 4 °C, rinsed in distilled water, dehydrated in a series of graded ethanol, passed through the transition solvent propylene oxide and then embedded in a mixture of Epon-812/Araldite. Serial thin sections (40–60 nm thick) were cut, contrasted with uranyl acetate and lead citrate, and observed with a Zeiss EM 109 (Carl Zeiss AD, Oberkochen, Germany).

## 3. Results

### 3.1. Light Microscopy

The animals used in this study were in the reproductive phase, as evidenced by the presence of all stages of spermatogenesis, including spermatozoa, in the seminiferous tubules and large secretory granules in the epithelial cells of the epididymal duct (Figure 2).

The longitudinal canal was located in the epididymal sheath (Figure 3). It was 600–700 µm long and had an irregular width, ranging from 47 µm to 120 µm.

This extratesticular canal was lined with a simple squamous epithelium, which became simple cuboidal epithelium near the openings of the efferent ducts (Figure 4A,B). The thickness of the epithelium increased (5.4 ± 0.6 µm vs. 9.5 ± 0.7 µm) near the efferent ducts, which were lined with a 27.10 ± 3.4 µm high simple columnar epithelium (Figure 4). The width of the lumen was irregular, ranging from 47 µm to 120 µm (mean: 74.50 ± 25.66 µm).

Cells from the testis were present in the lumen of the longitudinal canal (Figure 4A). The wall of this canal was devoid of a muscular layer and was surrounded by connective tissue (Figure 4A,B).

The efferent ducts, which originate from the longitudinal canal, were lined with high columnar cells (Figure 4).

The Alcian blue-PAS method revealed the presence of PAS-positive granules in the apical cytoplasm of the cells lining the longitudinal canal (Figure 5A,B). These granules were not visible in the epithelium of the efferent ducts (Figure 5B). Alcian blue did not stain any epithelial structures, whereas it faintly stained the connective tissue surrounding the longitudinal canal (Figure 5A).

### 3.2. Transmission Electron Microscopy

The epithelial cells of the longitudinal canal showed large, roundish euchromatic nuclei with prominent condensed nucleoli and apically located tight junctions (Figure 6A,B). The epithelium located distal to the opening of the efferent ducts consisted of non-ciliated cells, some of which were characterized by electron-dense granules located mainly in the apical region and by elongated and strongly electron-dense mitochondria scattered throughout the cytoplasm; very short microvilli were also present along the luminal border (Figure 6A). The presence of labyrinthine, narrow intercellular canaliculi was also observed (Figure 6A).

The epithelium lining the longitudinal canal near the opening of the efferent ducts consisted of two types of cells: non-ciliated cells and ciliated cells (Figure 6B). The non-ciliated cells exhibited the same ultrastructural characteristics described above, except for the presence of rod-shaped mitochondria. The ciliated cells had euchromatic nuclei containing clearly visible nucleoli. Their perinuclear cytoplasm was electron-lucent and contained cytoskeleton components (intermediate filaments and microfilaments), and the rod-shaped mitochondria had a less electron-dense matrix compared with non-ciliated cells.

The epithelium lining the efferent ducts had both ciliated and non-ciliated cells (Figure 7). In contrast to the longitudinal canal, the non-ciliated cells did not show secretory granules, whereas they displayed structures compatible with an absorptive phenotype such as numerous long microvilli, endosomes and lysosomes. Labyrinthine intercellular canaliculi detected in the longitudinal canal epithelium were absent.

## 4. Discussion

In squamate reptiles, sperm sequentially pass from the seminiferous tubules to the rete testis, ductuli efferentes, efferent ducts, ductus epididymis, ductus deferens and ampulla ductus deferentis [10,11,12,13,14]. In some Squamates, such as Anguidae *Anguis fragilis* [20], Scincidae *Plestiodon fasciatus* [21], and *Scincus scincus* [22] and in Lacertidae species such as *L. muralis* and *Lacerta agilis* [18,19,20,27], the extratesticular rete tubules (rete testis in other vertebrates), the number of which depends on species [12], empty into a longitudinal canal from which ductuli efferentes originate and pass into the epididymal ductus. Therefore, the longitudinal canal serves as an important passageway for sperm before they reach the epididymis, where maturation occurs.

Regarding the anatomical position of the longitudinal canal in Lacertidae, Padoa [18] reported that on the medial surface of the testis lies the hilum, the point of entry for blood vessels, nerve bundles and a single duct that connects the gonad to the head of the epididymis. The single extratesticular duct joins a longitudinal canal running along the outer margin of the epididymis. As in Lacertidae, a single extratesticular tubule opening into the longitudinal canal has been detected in the Scincidae *Plestiodon fasciatus* [21]. It has been reported that a single extratesticular tubule is an ancestral condition compared to a derived condition with multiple rete tubules [28]. This hypothesis is based on morphological and phylogenetic molecular studies [29,30].

As in other Lacertidae [18], we found that the longitudinal canal of *P. siculus* runs along the outer margin of the epididymis, and it was also close to the adrenal gland (Figure 3B). The proximity of the adrenal gland to the longitudinal canal is not surprising because in some vertebrates, including reptiles, the adrenal gland lacks a clear anatomical relationship with the kidney, being in close contact with the gonads and the genital ducts [29]. Among squamates containing a longitudinal canal, a topographical feature like *P. siculus* has also been detected in *Plestiodon fasciatus* [21] and *Scincus scincus* [22], in which the adrenal gland lies in the caput zone of the epididymis and a very thin layer of connective tissue separates the cortical strands of the adrenal from the caput epididymis.

The longitudinal canal has been largely ignored compared with the other structures constituting the accessory ducts of the testis, and there are few macroscopic and histological studies on this canal. A few studies reported the dimensions of this canal. In *Anguis fragilis*, the longitudinal canal is 3 mm long and 0.2 mm wide [20]. In *P. siculus*, this canal is 600–700 µm long and irregular in width, ranging from 47 µm to 120 µm. In *Lacerta* sp., the longitudinal canal is sometimes bifurcated with irregular enlargements along its course [17,20].

The results of this study are based on male specimens of *P. siculus* captured and euthanized during the breeding season, when the reproductive system reaches its maximum level of differentiation, and mature sperm production and mating occur.

The analysis of the histological sections revealed that the longitudinal canal of *P. siculus* is lined by simple squamous to low cuboidal epithelium that becomes simple cuboidal epithelium near the opening of the efferent ducts, which were characterized by a simple columnar epithelium. Its lumen often contained testicular-derived cells, including spermatozoa. A low epithelium has been observed in the longitudinal canal of the lizard *Lacerta muralis* [18]. In other reptiles, it has been reported that the longitudinal canal is lined by short cuboidal epithelium in *Plestiodon fasciatus* [21], by low epithelial cells in *Scincus scincus* [22], and by a simple squamous epithelium in *Scincella lateralis* [25]. The height of the epithelium in the longitudinal canal of *P. siculus* was very similar to that detected for *Plestiodon fasciatus* [21] (the only study to report the epithelial height during the reproductive period). Histomorphological staining such as Hematoxylin-Eosin and Galgano trichrome did not reveal the presence of secretory granules. This finding agrees with the lack of secretory activity observed in this canal by other authors using non-histochemical methods [17,18,20].

The histochemical investigation performed using the Alcian blue-PAS method revealed a lack of Alcian blue positivity in the epithelial cells and the presence of PAS-positive granules in the apical cytoplasm of the epithelial cells in the longitudinal canal of *P. siculus*. The lack of Alcian blue positivity indicates that epithelial cells of the longitudinal canal do not produce acidic glycans. The presence of apical PAS-positive granules demonstrates that this extra-testicular tract is capable of synthesizing neutral glycans, which may be secreted into the lumen. Therefore, it is reasonable to hypothesize that they may influence the chemical environment that sperm from the testes interact with as they travel towards the efferent ducts and, in turn, they may affect the glycocalyx composition of the spermatozoa. This hypothesis is supported by the increased levels of neutral glycans expressed in sperm from the efferent ducts [23] compared with testicular sperm [31] during the reproductive period of *P. siculus*. It should be noted that the ultrastructure of the efferent ducts of *P. siculus* does not show cytological features indicative of secretory activity [23]. Specifically, a lectin histochemistry study demonstrated an increase in glycoproteins containing mannose, galactose, and N-acetyllactosamine in the spermatozoa flowing in the lumen of the efferent ducts [23]. In mammals, it is well established that the glycocalyx of spermatozoa is modified during extratesticular transit and its glycan composition is critical for sperm survival in the female reproductive tract and sperm fertility [32]. A direct comparison with other reptiles is currently limited, as there are no similar histochemical studies of the longitudinal canal in other reptiles. However, in the proximal extratesticular ducts of other reptiles, a PAS-positive substance has been detected in the cytoplasm of the epithelium of the rete testis of the swamp snake (*Seminatrix pygaea*) [13] and the Mediterranean gecko *Hemidactylus turcicus* [12], but no PAS-positive secretory granules have been found. A negative histochemical reaction for acidic glycans has been reported in the above-cited reptile species [12,13].

The presence of secretory granules has been reported in the ducts connecting the branches of the rete testis to the efferent ducts of *Chalcides ocellatus* [22]. The author of that study referred to the connecting ducts as “Verbindungsstück”. The chemical composition of the secretory granules has not been investigated. In contrast to the rete testis, which has a squamous epithelium, these connecting ducts consist of a simple columnar epithelium characterised by the presence of secretory granules. Therefore, the Verbindungsstück cannot be regarded in the same way as the longitudinal canal, which is lined with a flat or cuboidal epithelium. The Verbindungsstück, in turn, opens into the efferent ducts, whose epithelium lacks secretory granules and is ciliated.

The present study examined, for the first time, the ultrastructure of the longitudinal canal of a reptile species. Transmission electron microscopy revealed that the epithelium lining the longitudinal canal of *P. siculus* consisted of non-ciliated cells and, near the openings of the efferent ducts, also contained ciliated cells.

The non-ciliated cells exhibited electron-dense granules in the apical region. Since apical granules were detected histochemically using PAS staining, it can be hypothesized that the epithelium lining the longitudinal canal of *P. siculus* may be involved in creating a biochemical environment that the sperm interact with as they pass towards the efferent ducts.

Other ultrastructural characteristics of the non-ciliated cells are the presence of labyrinthine intercellular canaliculi and short microvilli. The presence of intercellular canaliculi has been linked to the absorption of water and water-soluble particles by the epithelium [33].

The presence of short microvilli along the luminal border of the non-ciliated cells of *P. siculus* suggests that the longitudinal canal may be involved in the absorption of the luminal fluid components. The absorptive activity of the extratesticular rete testis in other vertebrates has been proposed [34].

Regarding the ciliated cells, their presence in the longitudinal canal has never been reported in any reptile species. This is because, as far as we know, no ultrastructural studies of the longitudinal canal have been carried out to date. This explains why Padoa [18] refers to a non-ciliated and non-glandular epithelium in the longitudinal canal of the wall lizard (*Lacerta muralis*). The presence of cilia and their occurrence near the junction of the longitudinal canal and the efferent ducts may be a result of their development from the mesonephric tubules, which, in the nephric portion of the tubule, contain ciliated cells [34]. However, occasional ciliated cells have been observed in the mouse rete testis epithelium at the junction between the rete testis and the efferent ducts [34]. The presence of ciliated cells and non-ciliated cells with short microvilli has been found in the epithelium of the extratesticular rete tubule in *Hemidactylus turcicus* [12]. The testicular spermatozoa are immotile and subsequently gain motility during transit through the ductus epididymis [35]. As the wall of the longitudinal canal lacks a muscular layer, the cilia may help in the transport of the sperm towards the efferent ducts as well as in generating a stirring movement of the luminal contents. In turn, the luminal fluid is absorbed into the efferent ducts, which, as we reported in the Results Section (Figure 7), are lined by a columnar epithelium, whose non-ciliated cells possess a well-developed microvillar apparatus and endocytic and lysosomal apparatus [36].

## 5. Conclusions

This study demonstrates that the epithelium lining the longitudinal duct of the epididymis in the lizard *P. siculus* can produce granules that are secreted into the duct lumen. Since the efferent ducts do not exhibit secretory activity and previous studies have shown higher glycan expression in sperm flowing through these ducts compared to those in the intratesticular ducts, the findings of the present study support the hypothesis that the epithelium of the longitudinal canal of the lizard *P. siculus* may influence the glycan composition of the sperm surface.

## Figures and Tables

**Figure 1:**
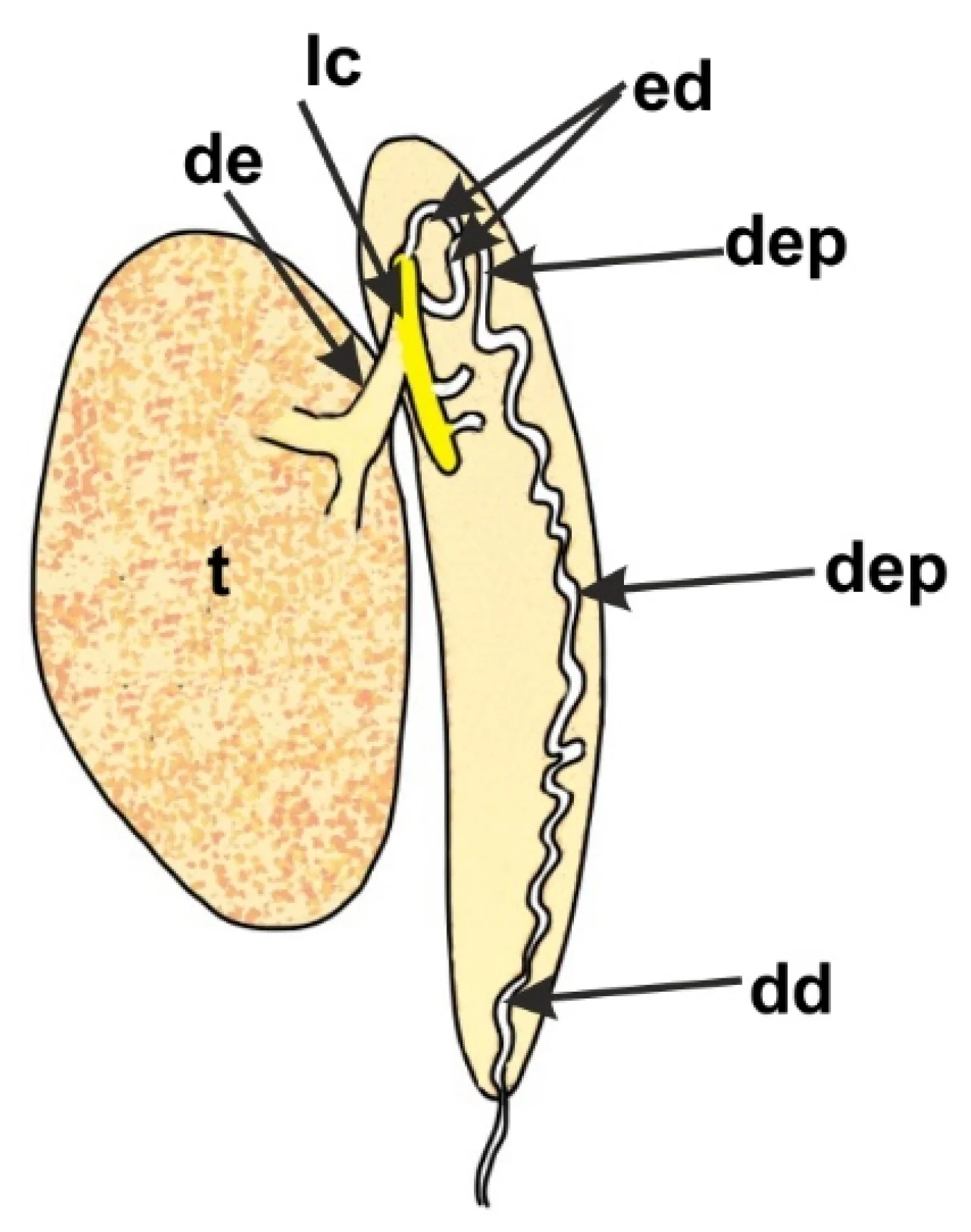
Schematic drawing of the testis-epididymis complex of the lizard *Podarcis siculus*, taken from a drawing by Fox [19] based on Alverdes [20] for four species of Lacertidae: *Lacerta agilis*, *Lacerta viridis*, *Podarcis muralis*, *Zootoca vivipara*. de, ductulus efferentis; dd, duct deferens; dep, ductus epididymis; ed, efferent ducts; lc, longitudinal canal; t, testis.

**Figure 2:**
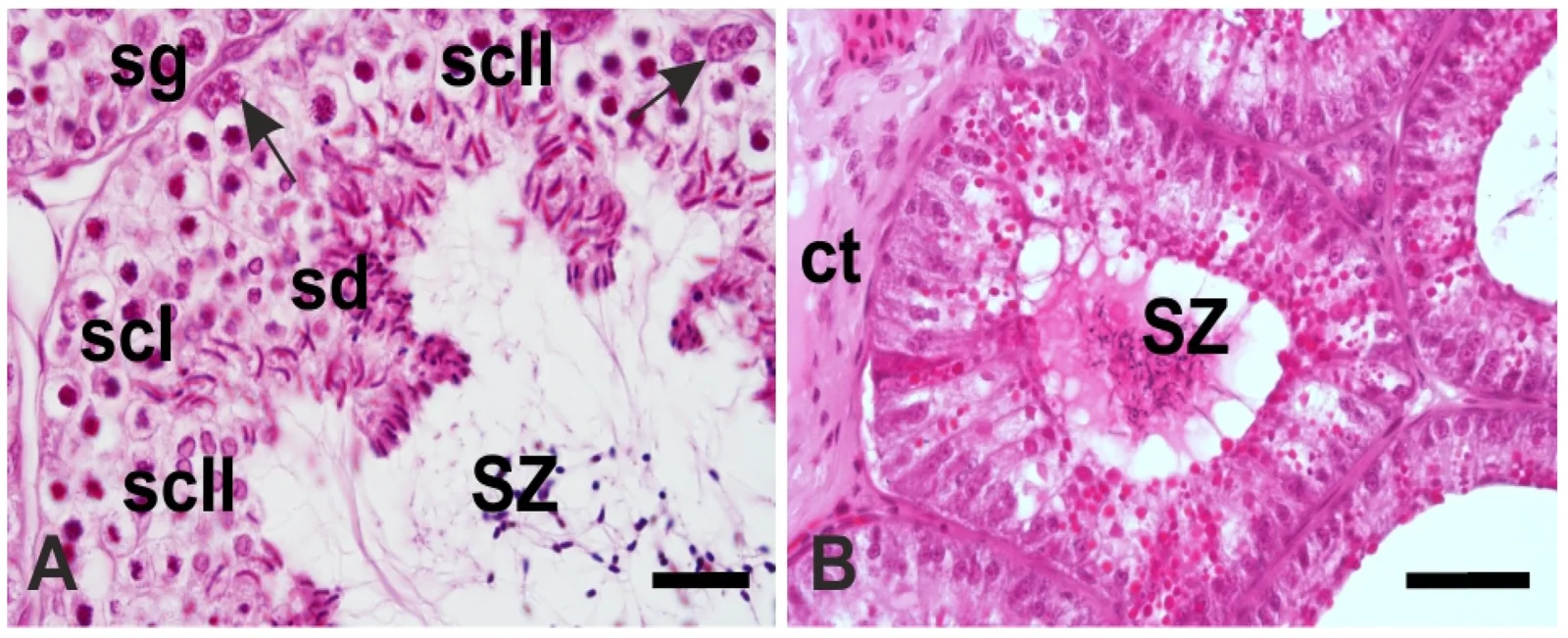
Details of the testis (**A**) and the epididymal duct (**B**) of *Podarcis siculus* during the reproductive period. Note the presence of all stages of spermatogenesis in the seminiferous tubules (**A**) and numerous secretory granules in the epididymal epithelium, with spermatozoa mixed with the luminal granules (**B**). ct, connective tissue; scI, spermatocytes I; scII, spermatocytes II; sd, spermatids; sg, spermatogonia; sz, spermatozoa; arrow, Sertoli cell. Hematoxylin-Eosin staining. Scale bar: (**A**) 20 µm; (**B**) 40 µm.

**Figure 3:**
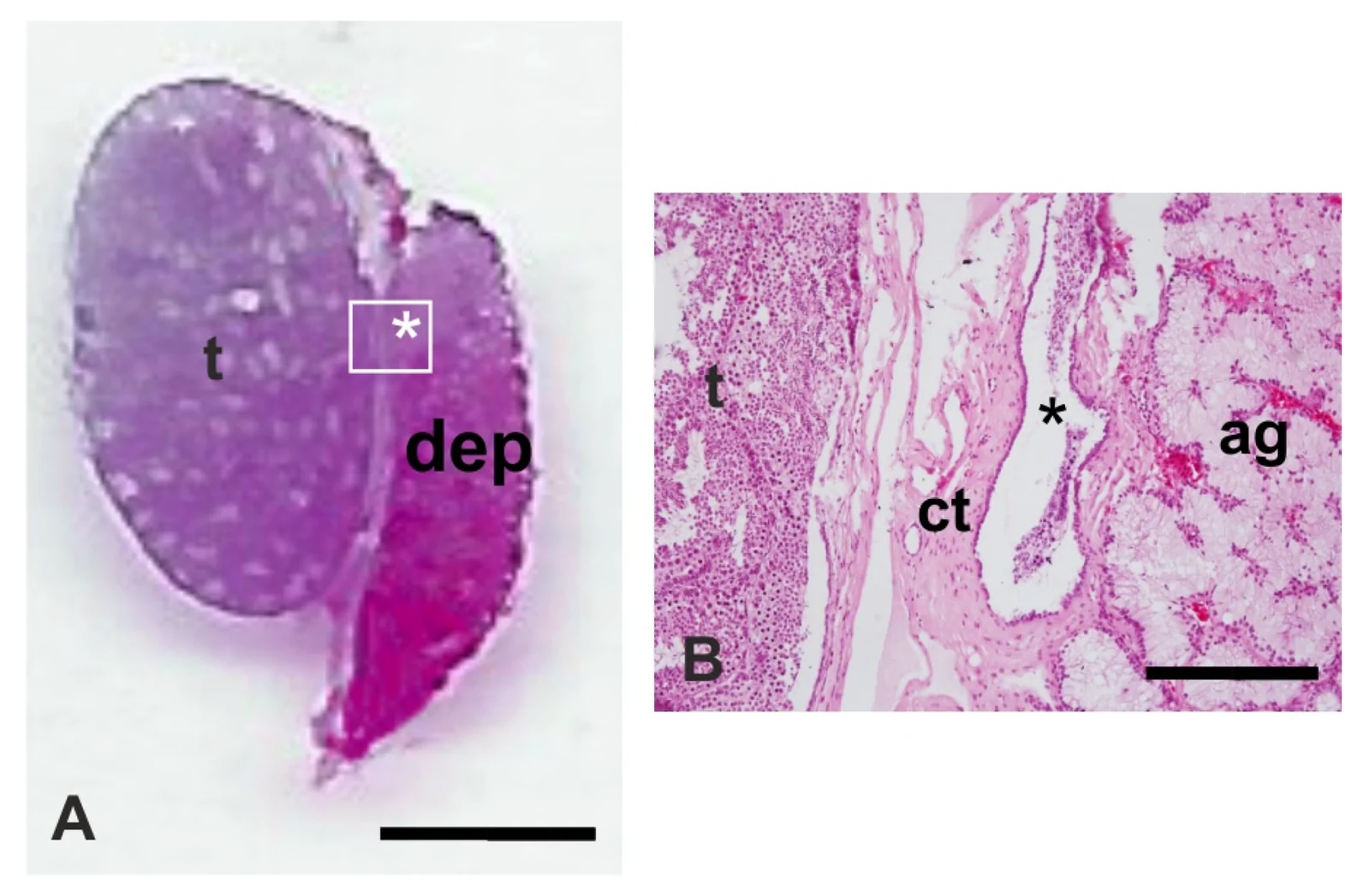
(**A**) Low-magnification view of a longitudinal histological section of the testis-epididymis complex of the lizard *Podarcis siculus* during the reproductive period. (**B**) Details of the boxed area of (**A**). Note the presence of testis-derived cells in the longitudinal canal. The adrenal gland, which is in proximity to the testis and the longitudinal canal is also visible. The adrenal gland of reptiles, with few exceptions, is near the gonads and genital ducts [26]. ag, adrenal gland; ct, connective tissue; dep, ductus epididymis; t, testis; asterisk, lumen of the longitudinal canal. Hematoxylin-Eosin staining. Scale bar: (**A**) 5 mm; (**B**) 200 µm.

**Figure 4:**
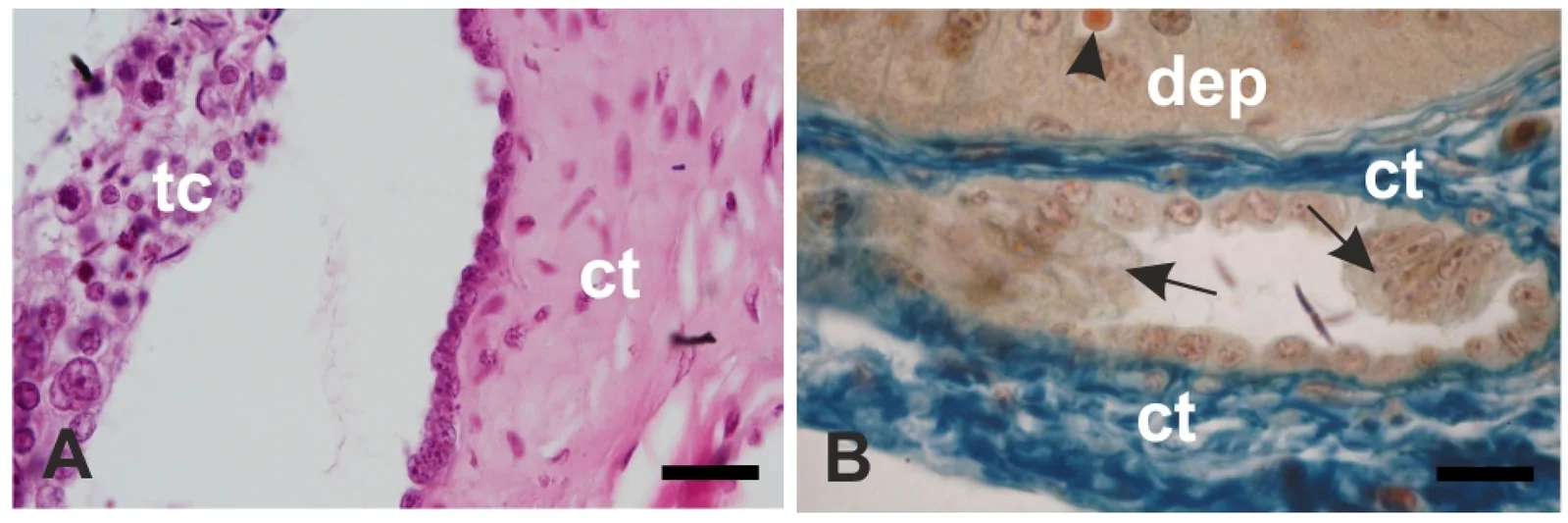
Details of the cranial part of the epididymis of *Podarcis siculus* showing the longitudinal canal distal to (**A**) and near to the opening of the efferent duct (**B**). Note the presence of testis-derived cells in (**A**), and columnar epithelium of the efferent duct in (**B**). ct, connective tissue; dep, ductus epididymis; tc, cells coming from testis; arrow, proximal efferent duct; arrowhead, granule in the epididymal cell. (**A**) Hematoxylin-Eosin staining; (**B**) Galgano trichrome staining. Scale bar: (**A**,**B**) 20 µm.

**Figure 5:**
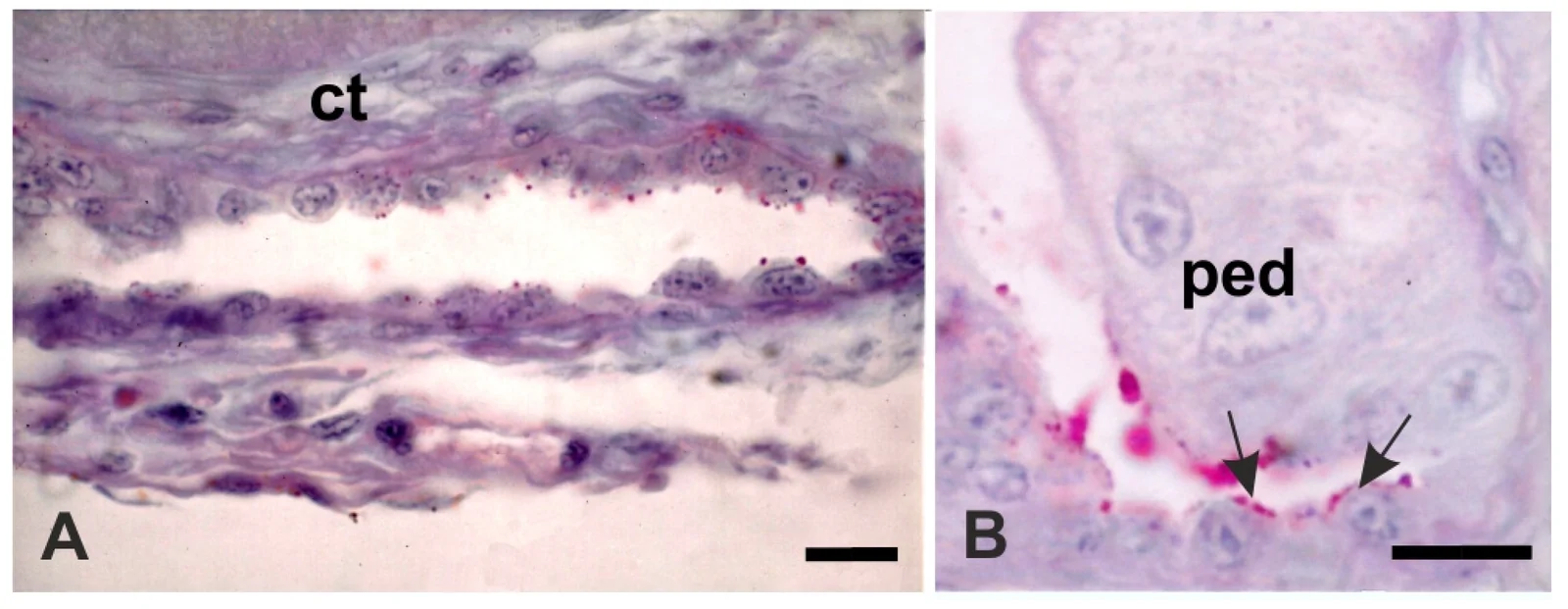
Alcian blue-PAS staining of the longitudinal canal (**A**) and the opening of a proximal efferent duct (**B**) of *Podarcis siculus*. Note the presence of PAS-positive apical granules in the longitudinal canal epithelium (**A**,**B**) and the absence of PAS staining in the cytoplasm of the efferent duct epithelium (**B**). Alcian blue faintly stained the connective tissue. ct, connective tissue; ped, proximal efferent duct; arrow, PAS-positive granules in cuboidal cells of the longitudinal canal. Scale bar: (**A**) 20 µm; (**B**) 40 µm.

**Figure 6:**
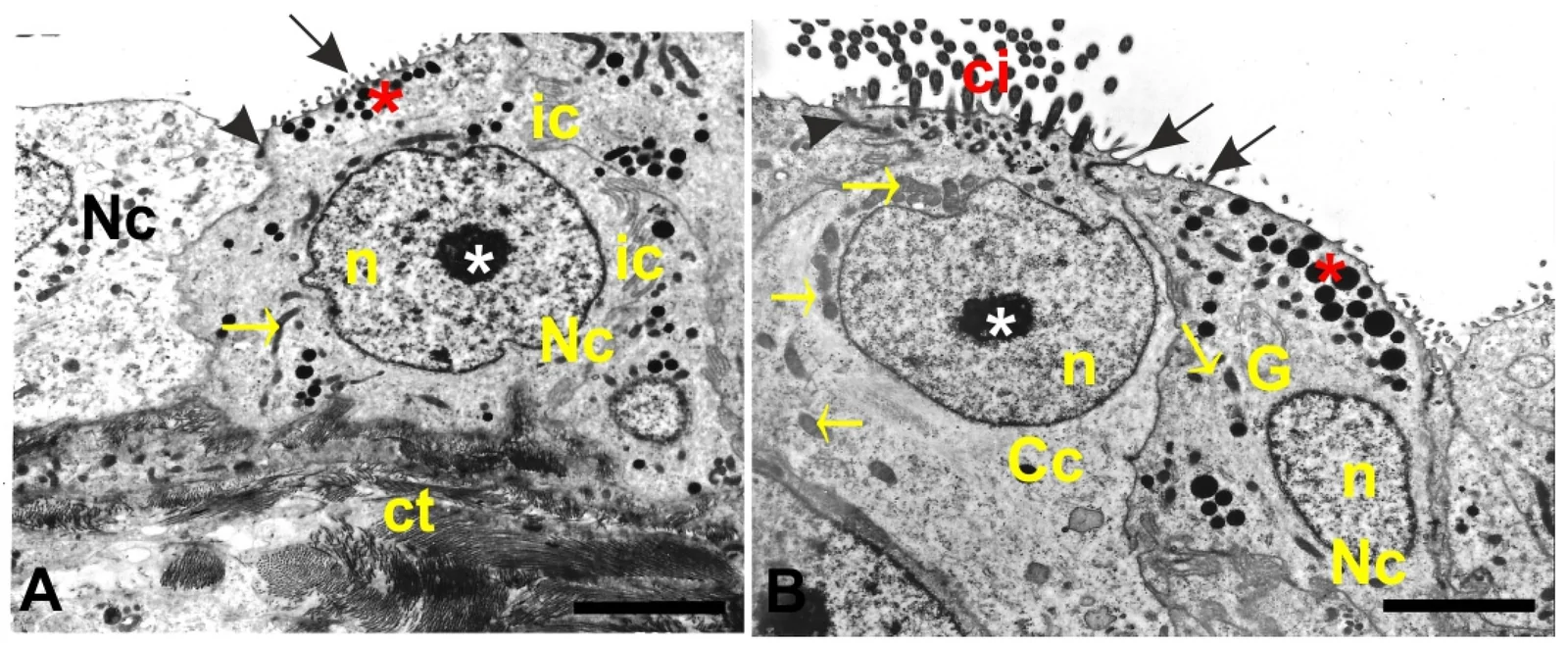
Transmission electron microscopy of the epithelium lining the longitudinal canal of *Podarcis siculus* distally (**A**) and near (**B**) the opening of the efferent ducts. Note the presence of electron-dense granules in the non-ciliated cells of both pictures and the presence of ciliated cells in the epithelium near the opening of the efferent ducts (**B**). Cc, ciliated cell; ci, cilia; ct, connective tissue; G, Golgi zone; ic, intercellular canaliculi; n, nucleus; Nc, non-ciliated cell; red asterisk, secretory granules; white asterisk, nucleolus; arrowhead, tight junction; black arrow, microvilli; yellow arrow, mitochondria. Scale bar: (**A**) 3 µm; (**B**) 4 µm.

**Figure 7:**
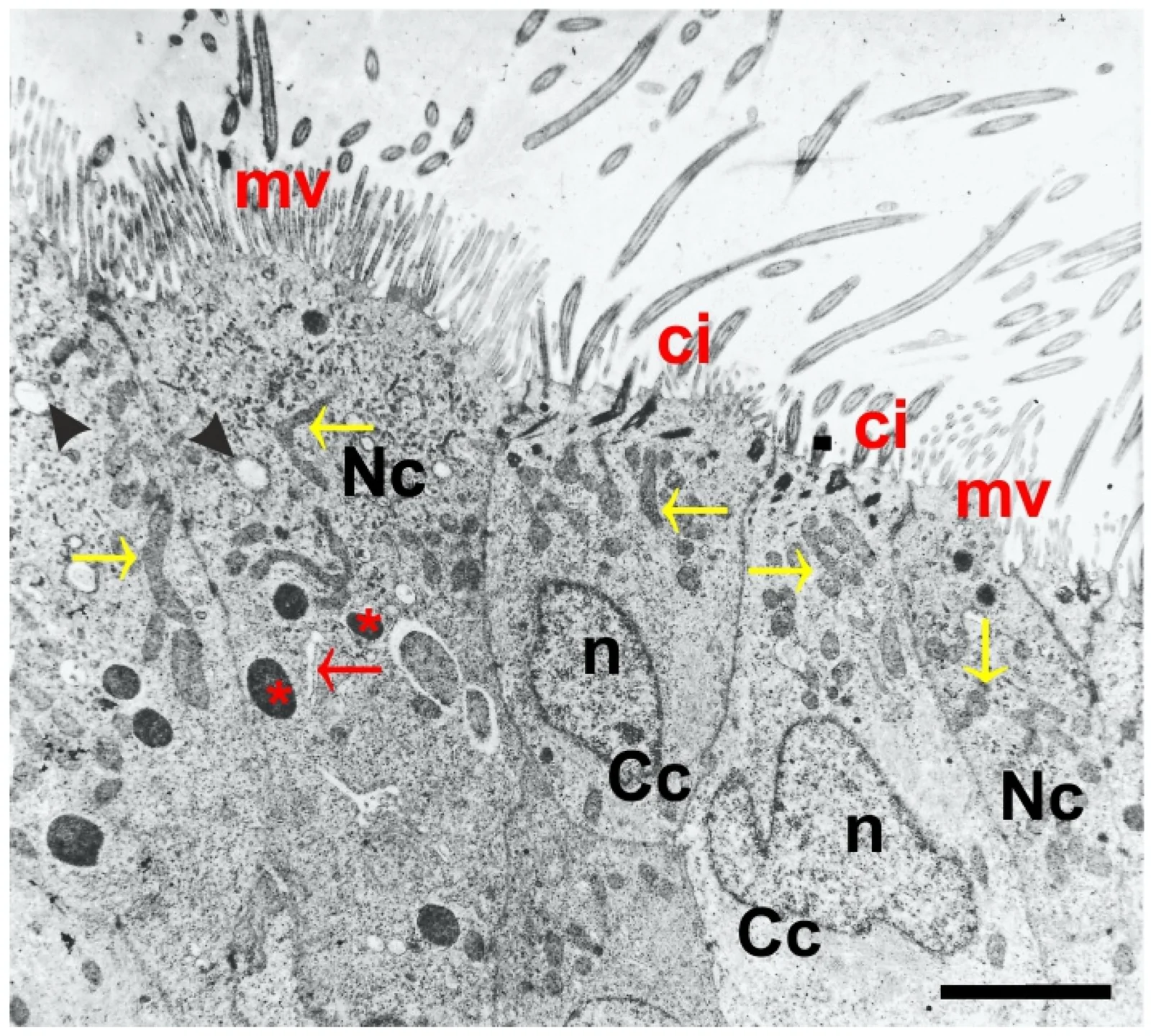
Ultrastructure of the epithelium lining the efferent ducts opening in the longitudinal canal of *Podarcis siculus*. Numerous long microvilli characterise the surface of the non-ciliated cells. Cc, ciliated cell; ci, cilia; Nc, non-ciliated cell; mv, microvilli; n, nucleus; yellow arrow, mitochondria; arrowhead, endosome; asterisk, lysosome; red arrow, Golgi zone. Scale bar: 4 µm.

## Data Availability

The original contributions presented in this study are included in the article. Further inquiries can be directed to the corresponding author.
